# Artificial Intelligence (AI) for Detection and Localization of Unobturated Second Mesial Buccal (MB2) Canals in Cone-Beam Computed Tomography (CBCT)

**DOI:** 10.3390/diagnostics12123214

**Published:** 2022-12-18

**Authors:** Lina Albitar, Tianyun Zhao, Chuan Huang, Mina Mahdian

**Affiliations:** 1 School of Dental Medicine, Stony Brook University, Stony Brook, NY 11794, USA; 2 Department of Biomedical Engineering, Stony Brook University, Stony Brook, NY 11794, USA; 3 Department of Radiology, Stony Brook Medicine, Stony Brook University, Stony Brook, NY 11794, USA; 4 Department of Prosthodontics and Digital Technology, School of Dental Medicine, Stony Brook University, Stony Brook, NY 11794, USA

**Keywords:** artificial intelligence, mesial buccal 2 canal, cone beam computed tomography, deep learning, MB2 canal, endodontics

## Abstract

The aim of this study was to develop a deep learning model to automatically detect and segment unobturated mesial buccal 2 (MB2) canals on endodontically obturated maxillary molars depicted in CBCT studies. Fifty-seven deidentified CBCT studies of maxillary molars with clinically confirmed unobturated MB2 canals were retrieved from a dental institution radiology database. One-hundred and two maxillary molar roots with and without unobturated MB2 canals were segmented using ITK-SNAP. The data were split into training and testing samples designated to train and evaluate the performance, respectively, of a convolutional neural network (CNN), U-Net. The detection performance revealed a sensitivity of 0.8, a specificity of 1, a high PPV of 1, and a NPV of 0.83 for the testing set, along with an accuracy of 0.9. The segmentation performance of unobturated MB2 canals, assessed using the custom metric, rendered a mean value of 0.3018 for the testing set. The current AI algorithm has the potential to identify obturated and unobturated canals in endodontically treated teeth. However, the AI algorithm is still somewhat affected by metallic artifacts, variations in canal calcifications, and the applied configuration. Thus, further development is needed to improve the algorithm and validate the accuracy using external validation data sets.

## 1. Introduction

The success rate of endodontic treatment is a public health issue that has economic and ethical repercussions. The success rate depends on a variety of factors, including the status of the pulpal and periodontal tissues, root canal anatomy, root canal shaping and obturation, and restorative treatment [1]. One of the most common attributable causes of endodontic failure is the failure to locate canals during endodontic treatment [2]. Variations in the existence and configuration of endodontic canals are dependent on age, ethnicity, and root anatomy, thus making their detection more complex [3,4]. Endodontically treated teeth with missed canals have a 4.4 times higher chance of having missed pathology due to loss of vitality of the tooth and the absence of symptoms [5]. 

Endodontic retreatment involves treating missed canals, with 93% of all missed canals being on the maxillary first molar and 44% on maxillary second molars [6,7]. Dentists use their knowledge of root anatomy [8] and radiographs [9] to help detect periapical pathology. While periapical radiographs are the primary imaging modality for endodontic diagnosis and treatment planning, cone beam computed tomography (CBCT) is the recommended diagnostic tool for complicated endodontic cases, including teeth with the potential for extra canals or as an intra-operative examination for identification and localization of calcified canals [10]. Numerous studies have documented the efficacy of CBCT for detecting a second mesiobuccal canal (MB2) in maxillary first and second molars, reported at 69.2–96.6% [11,12]. However, in reality, the presence of metallic restorations and endodontic obturation creates significant artifacts that limit the visualization of missed canals and render evaluations time-consuming and challenging for the general dentist and specialist with questionable accuracy [6,13]. 

Artificial intelligence (AI) is a collection of technologies that imitates intelligent human behavior to learn from experience [14]. A subset of AI, deep learning (DL), is comprised of multiple hidden layers of neural networks that mimic the function of biological neurons to progressively “learn” by extracting features from the input data [15]. Most of this implementation is centered on neural networks, such as convolutional neural networks (CNNs) [16,17]. With the introduction of AI in healthcare, DL is being used to streamline and supplement a practitioner’s knowledge in a wide range of areas, such as disease prediction and diagnosis and optimized management [15,18,19]. In dentistry, CNNs have been trained to extract data from images, such as alveolar crest bone level detection [20], dental caries detection [16], and pathology [21]. Fewer studies have evaluated AI in endodontics, a small proportion of which are based on three-dimensional volumetric data [22,23]. To date, research has not explored detecting unobturated canals in endodontically treated teeth with AI. Although a small number of recent studies have demonstrated the feasibility of identifying canals [23,24,25] using AI, these studies excluded scans with metallic artifacts. The aim of the present study was to develop a CNN to accurately detect unobturated canals in endodontically treated maxillary molars on CBCT compared to clinical and/or radiographic records of confirmed unobturated canals.

## 2. Materials and Methods

This study was approved by the Institutional Review Board (IRB2021-00085). A retrospective review of limited field of view (LFOV) CBCT scans (*n* = 400) acquired at the Stony Brook School of Dental Medicine for endodontic purposes was conducted, and CBCT studies of endodontically treated maxillary molars with unobturated MB2 canals were retrieved. Clinical endodontic confirmation of the presence of unobturated MB2 canal via review of electronic health records (EHR) or radiographic documentation of the presence of unobturated MB2 canal in the radiology report signed by a board-certified Oral and Maxillofacial Radiologist (OMFR) served as the ground truth. Inclusion criteria: LFOV CBCT scans of endodontically treated teeth with confirmed unobturated MB2 canals, with the Carestream 9000 and Carestream 9600 CBCT units (Carestream Dental LLC; Atlanta, GA, USA) at variable exposure settings compatible with the standard protocol for an average size patient, with kVps ranging between 70–120 kV, mA ranging between 3.2–10 mA, 19.0 s, and a voxel sizes of 0.76 and 0.75 mm for the Carestream 9000 and Carestream 9600, respectively. Metal artifact reduction (MAR) function available on Carestream 9600 was applied to scans, however no option for artifact reduction was available for scans acquired from Carestream 9000. Matching number of LFOV CBCT scans of endodontically treated teeth without unobturated canals were included. Exclusion criteria: scans acquired with voxel sizes greater than 0.76 mm; large field of view scans; scans with degraded image quality due to artifacts rendering poor visualization of the root canal system; duplicate scans of the same tooth.

Screening:

After the initial screening of the CBCT database and clinical records, all data were deidentified by removing protected health information (PHI), assigned an identifier (MB1, MB2, MB3, etc.) stored and processed within the secure and HIPAA compliant network of Stony Brook University Hospital for image analysis and data processing.

Fifty-eight CBCT scans, 41 acquired with the Carestream 9600 and 17 with the Carestream 9000, were used to locate one hundred and two roots with (*n* = 51) and without (*n* = 51) unobturated MB2 canals were included. An OMFR resident (LA) deidentified and imported the selected CBCT scans into an image segmentation software, ITK-SNAP [26] (open-source free software version 3.8.0) for manual segmentation of the root, the obturated, and the unobturated canals. The segmentations were reviewed by a board-certified OMFR (MM) for accuracy and stored in nifti format and shared with the AI team (CH and TZ) via the secure and HIPAA compliant network of Stony Brook University Hospital.

U-Net training and processing:

To train and test the network, the data were randomly split into training and testing with a ratio of 90:10. Roots from the same subjects were assigned to the same group. The training set was used to train and optimize the network model. The testing set was reserved for evaluation only. 

The original scan was cropped based on the manual root segmentation drawn by LA. So, only one root was visible in the image volume. Three-dimensional (3D) U-Net was chosen for this study to take advantage of the 3D information available. 

**Figure 1:**
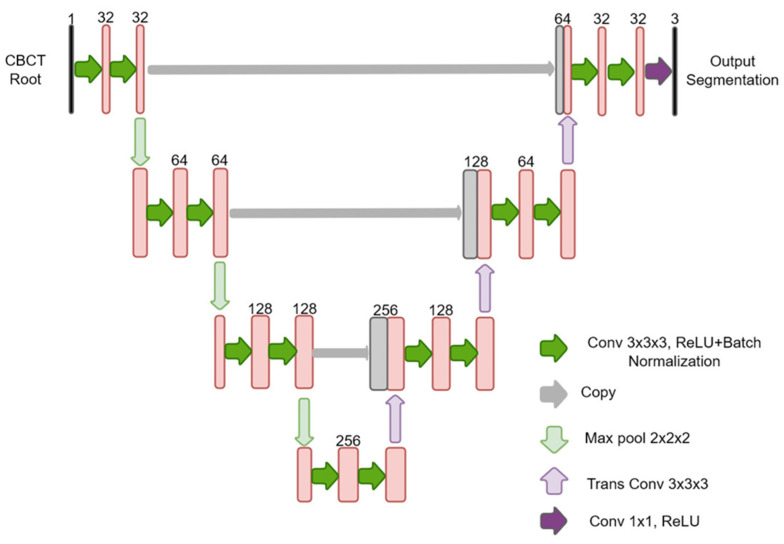
The U-Net architecture used in this study.

The dimension of the input image volume for the U-Net was (144, 144, 160). The input of each subject was normalized to be between 0 and 1 using min-max normalization based on the formula:

(I−min(V)/(max(V)−min(V)),

where *I* is the intensity of the voxel and *V* is the volumetric data containing the intensity of all voxels in the image. Data normalization was to ensure a similar range across all roots so the U-Net could be applied to all subjects. The employed U-Net architecture is shown in Figure 1. The inherent structure of the U-Net allows it to generate segmentation in the same resolution as the input image volume. The U-Net contains an encoding path, decoding path, and skip connection to provide robust and complex features to be observed by the network. The encoding path extracts features, while the decoder path project those features to the original input size to produce the masks. The skip connection helps increase network performance without requiring a deeper network. The skip connection concatenates features extracted by the encoder to allow localization of the feature [27]. Softmax activation function was used for the last layer to generate segmentation. 

During the training, the U-Net was set to learn the background, unobturated canals, and obturated canals. The loss function was sparse categorical cross-entropy, and the metric was the sparse categorical accuracy. The model was trained with Adam optimizer with an initial learning rate of 0.001. The learning rate was reduced to 0.33 of the previous rate if the metric did not improve for 80 epochs. The learning rate stopped reducing once it reached 0.00001, which helped the network to converge. The model training was set to terminate when the metric stopped improving for 100 epochs. The network parameters with the lowest loss were saved as the final model. The output of the network was a probability distribution predicting whether each voxel belonged to a class. The label with the highest probability was assigned to the voxel. The label would not be assigned if the probability was less than a certain threshold. The threshold was determined using the training data set.

Evaluation:

The trained model was applied to the independent testing data comprising five roots with and five without unobturated MB2 canals. The testing data were pre-processed in the same way as the training set to evaluate the performance of the model. The dice coefficient at the subject level is commonly used to assess the model’s segmentation performance using the formula [28]:

(2×area of mask overlap)/(area of manual mask+area of CNN mask),

However, in this study, the MB2 is small compared to the background. To fairly evaluate the performance of MB2 detection, we chose to use a custom metric to better evaluate the performance of the model. The custom metric is based on the Dice coefficient, and the formula is:

$$\frac{2\times \mathrm{slices}\;\mathrm{that}\;\mathrm{have}\;\mathrm{overlap}\;\mathrm{masks}}{\mathrm{total}\;\mathrm{slices}\;\mathrm{with}\;\mathrm{manual}\;\mathrm{mask}+\mathrm{total}\;\mathrm{slices}\;\mathrm{with}\;\mathrm{CNN}\;\mathrm{mask}}.$$

**Figure 2:**
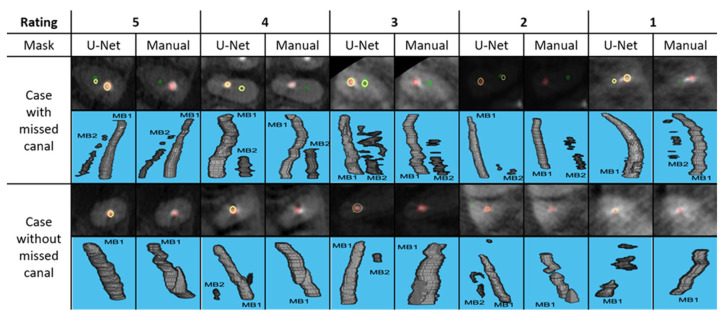
Typical examples of each segmentation rating containing CBCT axial cross section with segmentation overlaid (top) and 3D view (bottom). MB1 is obturated canal, depicted in red and MB2 is unobturated canal, depicted in green. The yellow ring represents the location of manual segmentation relative to U-Net generated segmentation.

In addition to the metric used above, a ranking system was developed for visual evaluation of the segmentation performance by two raters (TZ: technical expert, PhD student in biomedical engineering, LA: clinical expert, OMR resident) independently and any disagreement was resolved by a third evaluator i.e., tiebreaker (MM: clinical expert, board-certified OMR with seven years’ experience). Inter-rater agreement was calculated using the Cohen’s Kappa analysis. For the visual evaluation, the segmentation performance was rated from 1 to 5, with 5 being the best and 1 being the worst. Typical examples of each category are shown in Figure 2. For the evaluation of unobturated MB2 detection, similarly, three experts (TZ, LA, MM) compared the manual segmentation, and U-net generated segmentation to determine if the U-net segmentation is sufficient to determine the presence or absence of MB2. This was done independent of the custom metric and Dice coefficient.

## 3. Results

For detection performance, the model achieved a sensitivity of 0.8 and a specificity of 1 for the testing set, along with an accuracy of 0.9. The model also achieved a high PPV of 1 and a NPV of 0.83 for the testing set. The result is summarized in Table 1. 

**Figure 3:**
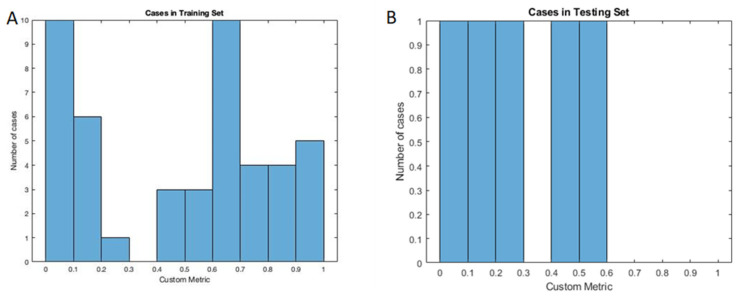
The histogram of the custom metrics between the manual segmentation and the U-Net generated segmentation for cases with unobturated MB2. (**A**) training set results; (**B**) testing set results.

**Figure 4:**
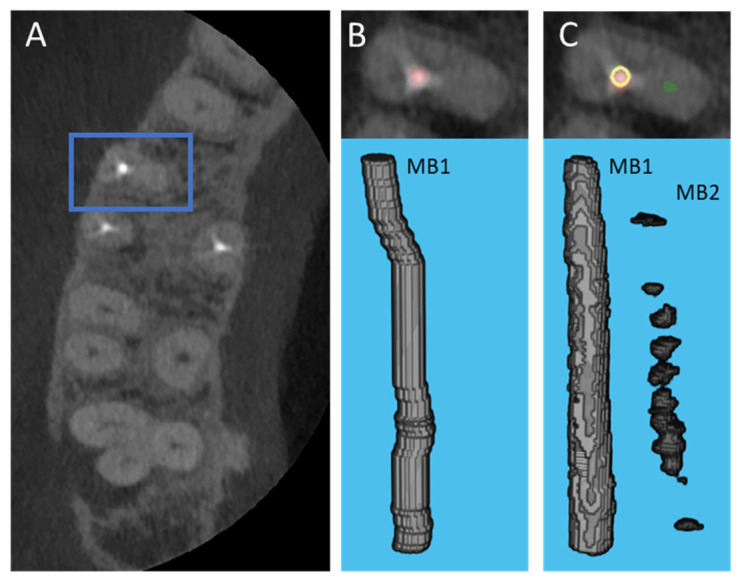
Case where U-Net performed better than the manual segmentation; an unobturated MB2 canal was present, yet the resident had difficulty segmenting the canal. (**A**) CBCT axial cross section view showing the location of the root, blue box indicates the root; (**B**) Manually generated segmentation visualized in the CBCT axial cross section (top) and the corresponding 3D view (bottom), the red color depicts the superimposed obturated MB1 canal; (**C**) U-Net generated segmentation visualized in the CBCT axial cross section (top) and the corresponding 3D view (bottom), the U-Net generated canal segmentation is red for the obturated MB1 canal and green for the unobturated MB2 canal, the yellow ring represents the location of manual segmentation relative to U-Net generated segmentation.

The segmentation performance was quantitatively assessed using the custom metric adapted from the Dice coefficient. The custom metric for cases with unobturated MB2, the U-net model achieved a mean value of 0.4724 (95% CI: 0.3731–0.5717) for the training set and 0.3018 (95% CI: 0.0388–0.5649) for the testing set. The distribution of the custom metric is shown in Figure 3. The expert rating result for cases with and without unobturated MB2 canals is shown in Table 2, and the segmentation rating shows promising results. The model’s segmentation performance was better in cases without unobturated MB2 cases. This means the models will produce fewer false positives in terms of detection. The evaluation of the detection performance confirmed this. In some cases, the U-Net even outperformed manual segmentation. One example is shown in Figure 4, whereby the case was deemed as having unobturated MB2 by the resident based on the root anatomy, location of the obturated canal, and presence of periapical pathology on the palatal aspect of the root, but the canal could not be manually segmented due to beam hardening artifacts and canal configuration using the CBCT images. U-Net successfully identified the unobturated MB2 canal and was validated by the clinicians.

Inter-rater agreement of the segmentation ranking was divided between roots with and without unobturated MB2 canals. Of the 102 cases, 44 needed a tiebreaker. The inter-rater reliability in evaluating the segmentation performance for the MB2 cases were as follows: LA vs. TZ: Kappa = 0.346 (*p* < 0.00), 95% CI (0.174, 0.518), with fair agreement 50.9% of the time. Tiebreaker vs.TZ: Kappa = 0.64 (*p* < 0.00), 95% CI (0.480, 0.800), with moderate agreement between the two raters, 72.5% of the time. Tiebreaker vs. LA: Kappa = 0.6 (*p* < 0.00), 95% CI (0.432, 0.762), with moderate agreement 70.5% of the time. The inter-rater reliability in evaluating the segmentation performance for the non-MB2 cases were as follows: LA vs. TZ: Kappa = 0.314 (*p* < 0.00), 95% CI (0.14, 0.488), with fair agreement 64.7% of the time. Tiebreaker vs. TZ: Kappa = 0.456 (*p* < 0.00), 95% CI (0.26, 0.652), with moderate agreement between the two raters, 76.4% of the time. Tiebreaker vs. LA: Kappa = 0.729 (*p* < 0.00), 95% CI (0.549, 0.900), with substantial agreement 86.2% of the time.

## 4. Discussion

The results of this pilot study demonstrate the following: (1) The deep learning algorithm demonstrated a progressively improving performance in terms of the sensitivity of detecting unobturated MB2 canals from the training phase (70%) to the testing phase (80%) and an overall acceptable specificity (98%), for the detection task; and (2) Evaluating the segmentation performance of the algorithm with a custom metric, the model achieved a mean value of 0.4724 for the training set and 0.3018 for the testing set. These results show that the model has a low chance of producing a false-positive result, which means a low probability of unnecessary treatment. 

The segmentation performance was satisfactory, given the nature of the problem. The custom metric cannot effectively evaluate the performance of the U-Net on cases without unobturated MB2, as there is no manual segmentation for MB2. In many cases, the resident found it difficult to segment the canal on all slices as it was challenging to distinguish the MB2 from the background due to beam hardening artifacts, anatomical configuration, and calcification of the canal. The custom metric used in this work penalizes the cases where U-Net’s segmentation are larger than the manual ones. This is ideal for evaluating the cases with perfect manual segmentation. However, it does not fully represent the performance of the U-Net due to the limitation of the metric. As shown in Figure 4, the custom metric in both cases was 0, but U-Net actually picked up the unobturated MB2 while a human could not, due to low resolution and contrast. Hence, the expert visual rating of the segmentation is more representative of segmentation performance. The model performed well in cases without unobturated MB2. A rating of 5 for cases without unobturated MB2 was assigned to thirty-two out of the 46 cases in training, and 3 out of the 5 testing cases. Although the result for cases with unobturated MB2 was not as good, the model’s performance was promising, as 35 out of the 46 cases were rated as 3 or higher in the training set, and 4 out of the 5 cases were rated as 3 or higher in the testing set. In some cases, the U-Net’s output is better than the human, as shown in Figure 4. 

The visual ranking of segmentation performance was completed independently by (1) TZ, a non-clinical expert, (2) LA, a clinical expert, and (3) Tiebreaker, (MM) a board-certified OMFR. Of the 102 cases, 44 needed a tiebreaker. Overall, the segmentation performance was ranked higher by the clinicians (LA and MM) likely influenced by the clinical knowledge of the canal anatomy, whereas for the non-clinical expert, the evaluation of segmentation performance is more based on pixel overlaps and less identification of anatomy. Likewise, a greater agreement was observed between the clinicians in segmentation rankings for the MB2 group. The overall inter-rater agreement between the non-clinical and clinical experts improved in cases without unobturated MB2 canals, as the model performed consistently better in segmenting the obturated canal in this group (better specificity). This study was the first to demonstrate a promising performance for a deep CNN to detect and localize unobturated MB2 canals in endodontically treated maxillary molars in CBCT data set and in the presence of metallic artifacts. A probabilistic neural network in Johari et al. (2017) evaluated vertical root fractures in vital and endodontically treated teeth, without coronal restorations using CBCT and intraoral periapical radiographs. As one of the few studies evaluating teeth with obturations in CBCT, they proved that the probabilistic neural network diagnosed vertical root fractures using CBCT scans more effectively than periapical radiographs, and ultimately a good model for assisting clinicians in detecting vertical root fractures [29]. In another study by Jeon et al. (2021), the detection of C-shaped canal anatomy was tested and compared to three different DL architectures (U-Net, residual U-Net, and Xception U-Net). Data collection included CBCT studies of 135 non-restored C-shaped mandibular molars, 100 of which were randomly used for training and validation, and the last 35 were for testing. The results were promising, with Dice coefficient scores of 0.768 for Xception U-Net, 0.76 for residual U-Net, and 0.66 for U-Net [30].

Duan et al. (2021) evaluated accurate unobturated tooth and pulp cavity segmentation using a two-phase deep learning technique; the first phase used CBCT reconstructed panoramic radiographs to isolate single and multirooted teeth using a Region Proposal Network (RPN) and Feature Pyramid Network (FPN) and those refined segmentations would be used within U-Net. Dice coefficient values were very high single and multirooted teeth, 95% and 96%, respectively, and slightly lower for the pulp cavity, 88% (single) and 86% (multirooted) [24].

In clinical practice, beam hardening artifacts associated with the presence of metallic restorations and endodontic obturations are an inevitable source of noise in CBCT scans [10,31]. Additionally, unobturated canals commonly present with significant calcifications and variations in the anatomical configuration, which combined with the metallic artifacts, makes their identification an extremely challenging task with questionable accuracy and an increased likelihood of false-positive diagnosis both for the clinician and AI. Our approach is unique in not only striving to evaluate unobturated canals in teeth, but to replicate the reality and challenge of what the clinician sees routinely on restored teeth. This pilot study presented with numerous limitations and challenges impacting the performance of the algorithm and interpretation of the results. One of the key factors affecting the success of a deep learning model is the sample size. Fang et al. (2021) conducted a study to evaluate the optimum sample size to train a deep learning algorithm for the segmentation of multiple regions of interest in the head and neck such as optic nerves, parotid glands, and brainstem. It was found that smaller organs such as lens of the eye or the optic nerve required 200 samples for 95% effectiveness, yet larger organs, such as the brainstem, temporal lobe, required 40 samples to achieve 95% of the best performance [32]. Given the complexity of the segmentation task and smaller region of interest in the present study, a larger and more heterogenous data set is desired for improved performance of the model. Detection and localization of unobturated MB2 canals is inherently a challenging task that is limited by variations in canal morphology, calcifications, and CBCT artifacts. Clinicians’ experience is also an important consideration in identifying these canals. In the present study, one evaluator (LA, OMR resident) with basic training in identifying these canals under the supervision of an experienced board-certified OMR (MM) performed manual segmentation of the canals, with limited ability to correct the planes along the long axis of the roots for better visualization of the canals. This limitation affects the accuracy of manual segmentation. Having an expert panel of 2–3 investigators with a radiology background and experience in localizing unobturated MB2 canals in CBCT studies to verify the accuracy of detection and segmentation would enhance the reliability of the training data set. The manual segmentation was also affected by the limitation of the ITK-SNAP program, with its inability to correct the planes of the CBCT, a valuable tool to view the canal from all angles when assessing for a partially calcified MB2 canal. By not being able to correct the planes, segmenting is more tedious with a higher chance of error, especially in tortuous or calcified canals. 

Finally, testing the algorithm on a variety of deep learning models or different CBCT machines would provide stronger, more universal results. Our current AI algorithm is still somewhat affected by metallic artifacts, variations in canal calcifications, and the applied configuration. Thus, further development is needed to improve the algorithm and validate its accuracy using external validation data sets. 

## 5. Conclusions

AI has the potential to identify obturated and unobturated canals in endodontically treated teeth. The current AI algorithm is still somewhat affected by metallic artifacts, variations in canal calcifications, and the applied configuration. Thus, further development is needed to improve the algorithm and validate the accuracy using external validation data sets.

## Figures and Tables

**Table 1:** Performance metrics of the model for the detection task in the training set and testing set.

	Sensitivity	Specificity	NPV	PPV	Accuracy
Training Set	0.70	0.98	0.76	0.97	0.84
Testing Set	0.80	1	0.83	1	0.90
All Data	0.71	0.98	0.76	0.97	0.84

**Table 2:** Rating of the segmentation performance of the U-Net model.

	Training (# of Cases)		Testing (# of Cases)	
Segmentation Rating	Cases with Unobturated MB2	Cases without Unobturated MB2	Cases with Unobturated MB2	Cases without Unobturated MB2
5	9	32	0	3
4	18	12	1	2
3	8	2	3	0
2	6	0	1	0
1	5	0	0	0

## Data Availability

The data presented in this study are available on request from the corresponding author. The data are not publicly available due to it being part of an academic institution.
